# 10-Acetylirciformonin B, A Sponge Furanoterpenoid, Induces DNA Damage and Apoptosis in Leukemia Cells

**DOI:** 10.3390/molecules171011839

**Published:** 2012-10-09

**Authors:** Jui-Hsin Su, Wen-Been Chang, Huei-Mei Chen, Mohamed El-Shazly, Ying-Chi Du, Ting-Hsuan Kung, Yu-Cheng Chen, Ping-Jyun Sung, Yuan-Shing Ho, Fu-Wen Kuo, Mei-Chin Lu

**Affiliations:** 1 Graduate Institute of Marine Biotechnology, National Dong Hwa University, Pingtung 944, Taiwan; 2 National Museum of Marine Biology & Aquarium, Pingtung 944, Taiwan; 3 Institute of Marine Biodiversity and Evolution, National Dong Hwa University, Pingtung 944, Taiwan; 4 Department of Nutrition, Lee’s Endocrinology Clinics, Pintung 900, Taiwan; 5 Graduate Institute of Natural Products, College of Pharmacy, Kaohsiung Medical University, Kaohsiung 807, Taiwan; 6 Department of Pharmacognosy and Natural Products Chemistry, Faculty of Pharmacy, Ain-Shams University, Organization of African Unity Street, Abassia, Cairo 11566, Egypt; 7 Eastern Marine Biology Research Center, Fisheries Research Institute, Taitung 961, Taiwan

**Keywords:** 10-acetylirciformonin B, apoptosis, DNA damage, sponge

## Abstract

10-Acetylirciformonin B, a furanoterpenoid derived from irciformonin B found in a marine sponge, has been reported to possess potent cytotoxic activity against several cancer cell lines. However, the mechanism of its apoptotic activity against human leukemia cells has never been reported. The purpose of this study was to investigate the cytotoxic effects of 10-acetylirciformonin B and its possible mechanism of action against leukemia HL 60 cells. We found that 10-acetylirciformonin B decreased cell viability through the inhibition of cell growth as well as the induction of DNA damage and apoptosis in a dose-dependent manner. The induction of DNA damage was mediated by the increase of p-CHK2 and γ-H2A.X, which was suggested from the increase of tail movement in the neutral Comet assay. Induction of apoptosis was mediated with the increase in caspases 8, 9 and 3 activation as well as PARP cleavage. In summary, our resultsindicate that 10-acetylirciformonin B treatment causes apoptosis in leukaemia cells; probably through a caspase-dependent regulatory pathway.

## 1. Introduction

The development of anticancer agents with selective toxicity to cancer cells with minimum or no toxicity to normal cells is still an unmet goal [1,2,3,4]. Recently some DNA damaging agents have emerged as potent selective cytotoxic agents with minimum toxicity to normal cells [5,6,7]. Tumor cells generally suffer from genomic instability and genomic defects. Thus, tumor cells lack appropriate responses to DNA damage [8]. Following the induction of DNA damage, a prominent apoptosis route is triggered in tumor cells [9]. These routes include O6-methylguanine, base *N*-alkylation, bulky DNA adducts, DNA cross-links and DNA double-strand breaks (DSBs). Most studies have pointed out that the induction of DSBs triggers phosphorylation of H2A.X (-H2A.X) that is mediated by ataxia telangiectasia mutated (ATM) and ataxia telangiectasia and Rad3 related (ATR) proteins, which signal downstream to CHK1, CHK2 (checkpoint kinases) and p53 [10,11,12]. In mammalian cells, it has been shown that CHK1 and CHK2 play a central role in transducing DNA damaging signals following prompted cell cycle checkpoints [6,13]. The activation of ATM upon DNA damage leads to the phosphorylation and activation of downstream effectors. Among the downstream targeted substrates are p53, CHK1, CHK2 and H2A.X. This activation of downstream effectors is critical as it is involved in regulation of multiple cellular processes including cell cycle progression [14]. As most clinical anti-cancer drugs target DNA, increasing knowledge on DNA damage-triggering signals leading to cell death is expected to provide new strategies for therapeutic interventions [14].

**Figure 1:**
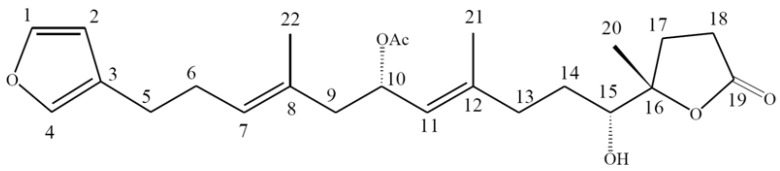
Chemical structure of 10-acetylirciformonin B isolated from marine sponge *Ircinia* sp.

Chemical substances derived from animals, plants and microbes have been utilized to treat human diseases since the dawn of reported history [15,16,17]. Marine sponges are a “gold mine” for potential therapeutic agents with respect to the diversity of marine organisms as well as the unprecedented diversity of their secondary metabolites discovered during the past fifty years [15,16,17,18,19]. In recent years, a variety of marine organisms have proven to be extremely potent against an increasing list of life threatening diseases, including cancer, Alzheimer’s disease and atherosclerosis. Several studies have shown that marine sponge metabolites possess strong selectivity, not only for specific kinases, but also for cancerous cells over healthy cells and thus represent promising candidates in the development of new oncology drugs [19]. As part of our natural products screening program for new anticancer lead compounds, ircinolin A, irciformonin B, irciformonin F, 15-acetylirciformonin B and 10-acetyl- irciformonin B were isolated from the marine sponge *Ircinia* sp. and exhibited potent cytotoxicity against K562, DLD-1, HepG2, and Hep3B cancer cell lines [20]. Among the isolates, 10-acetylirciformonin B (Figure 1) exhibited the highest potential activity against several cancer cell lines [20]. Encouraged by the aforementioned results the related cytotoxic mechanism of 10-acetylirciformonin B against leukemia HL 60 cells was investigated and the results are reported in this study.

## 2. Results and Discussion

### 2.1. 10-Acetylirciformonin B is A Potential Inhibitor of Cell Growth and Inducer of Apoptosis in Leukemia HL 60 Cells

**Figure 2:**
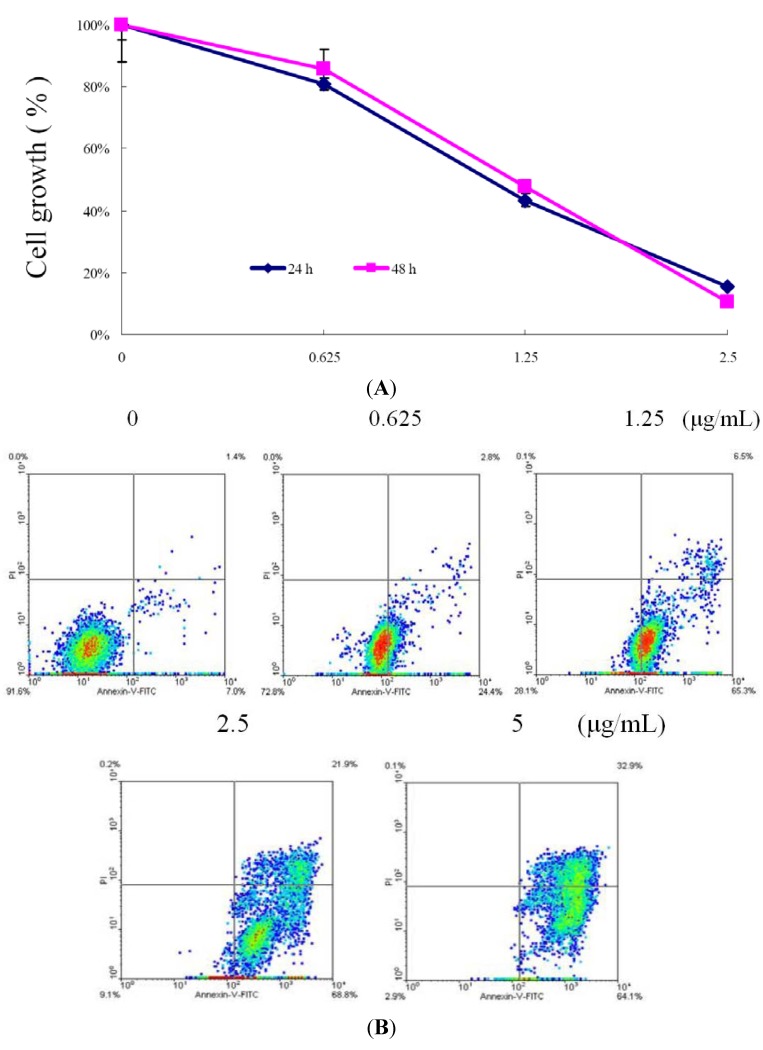
Cytotoxic and apoptotic effect of 10-acetylirciformonin B on HL 60 cells. (**A**) HL60 cells were treated with varying concentrations of 10-acetylirciformonin B for 24 and 48 h. Cell viability was evaluated by MTT assay. (**B**) HL 60 cells were treated with varying concentrations of 10-acetylirciformonin B for 24 h then labeled with annexin V-FITC and PI (propidium iodide) and analyzed with flow cytometry.

Linear C22-sesterterpenoids from the marine sponge *Ircinia* sp. were isolated, purified and studied for their growth inhibitory effect against different cancer cells in our previous study [20]. The strong cytotoxic activity of 10-acetylirciformonin B against human leukemia HL 60 cells suggested the need to study its cytotoxic mechanism(s) as a crucial step for its further development as a potential anticancer agent. The effect of 10-acetylirciformonin B on the growth of human leukemia HL 60 cells was determined using an MTT assay. After the treatment with 10-acetylirciformonin B for 24 and 48 h, growth of cancer cells were markedly inhibited in a dose- and time-dependent manner as compared to the control (Figure 2A). 

The calculated IC_50_ values of 10-acetylirciformonin B were 1.8 and 1.7 g/mL at 24 and 48 h, respectively. To evaluate whether the cytotoxicity of 10-acetylirciformonin B was associated with the induction of apoptosis, annexin V-FITC and propidium iodide (PI) staining assays were used. As shown in Figure 2B, treatment with 10-acetylirciformonin B at concentrations of 0, 0.625, 1.25 and 2.5 g/mL, increased the percentages of annexin-positive cells from 7% to 97% in a dose-dependent manner, indicating that 10-acetylirciformonin B treatment induces apoptosis in HL 60 cells. 

### 2.2. 10-Acetylirciformonin B Treatment Induced HL 60 Cells DNA Double-Strand Breaks

To examine if the antiproliferative and the apoptotic effect of 10-acetylirciformonin B involve induction of DNA strand breakages (DSBs) in human leukemia HL 60 cells, a Comet assay under neutral electrophoresis conditions was utilized.

**Figure 3:**
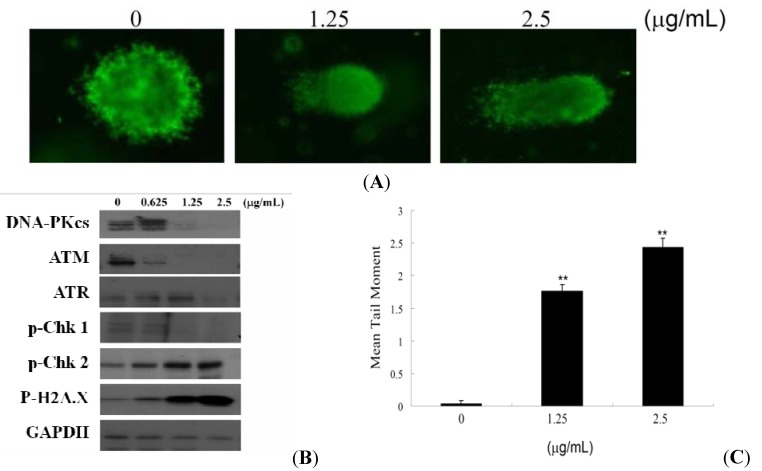
Effect of 10-acetylirciformonin B on the induction of double-strand breaks in HL 60 cells. (**A**) An example of “comet tail” due to chromosomal DNA double-strand breaks in 10-acetylirciformonin B (1.25 and 2.5 μg/mL)-treated HL 60 cells compared to the untreated control. Electrophoresis was carried out under neutral conditions. (**B**) Cells were harvested and lysates were prepared and subjected to SDS-PAGE followed by immunoblotting for DNA damage-related proteins. GAPDH was used as the loading control. (**C**) Quantitative results showing a gradual increase in tail moment upon 10-acetylirciformonin B treatment when compared with the control. Results are presented as mean ± SD of three independent experiments (* *p* < 0.05).

Different concentrations of 10-acetylirciformonin B (0, 1.25, and 2.5 µg/mL) for 24 h were tested and the level of nuclear DNA integrity was analyzed. As shown in Figure 3A,C, 10-acetylirciformonin B at 1.25 and 2.5 µg/mL increased the degree of DNA migration in HL 60 cells. The DNA migration was represented by the increase of DSBs in a dose-dependent manner, as indicated by abnormal tails’ sizes in the Comet assay. 10-Acetylirciformonin B caused DSBs, leading to the activation of cell cycle checkpoints in HL 60 cells which was suggested by the phosphorylation of CHK2 and H2A.X (Figure 3B). Treatment with different concentrations of 10-acetylirciformonin B at 24 h resulted in the phosphorylation of H2A.X at serine 139 (γ-H2A.X) and p-CHK2 at threonine 68 indicating a strong nuclear DNA damage (Figure 3B).

### 2.3. 10-Acetylirciformonin B Induced HL 60 Cells Apoptosis through Caspase-Dependent Pathway

**Figure 4:**
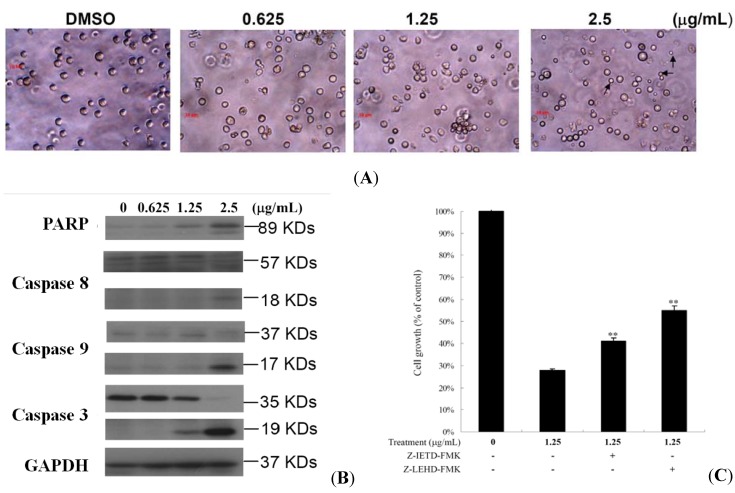
Apoptotic effect induced by 10-acetylirciformonin B in HL 60 cells. (**A**) Cells were treated with 0.625, 1.25 and 2.5 g/mL of 10-acetylirciformonin B for 24 h, and photographed under microscope (400×). The arrowheads point at the apoptotic bodies; (**B**) Western blotting analysis of apoptosis-related proteins expression in HL 60 cells after 10-acetylirciformonin B treatment at different concentrations for 24 h. GAPDH was used as an internal control to show the equal loading of protein; (**C**) HL 60 cells were preincubated with 25 nM caspase 8 or 9 inhibitors (z-DEVD-fmk or z-IETD-fmk) for 2 h, followed by 10-acetylirciformonin B treatment for 24 h. Cell viability was evaluated by MTT assay. Untreated cells were used as a control.

Morphologically apoptotic cells in 10-acetylirciformonin B-treated HL 60 cells were characterized by the formation of apoptotic bodies (Figure 4A apoptotic induction, we investigated the expression of apoptosis-related proteins in 10-acetylirciformonin B treated HL 60 cells using a Western blotting assay. As shown in Figure 4B, treatment of HL 60 cells with 10-acetylirciformonin B for 24 h increased the activation of caspases 3, 8 and 9 as well as the cleavage of PARP (89 KDa) in a dose-dependent manner. In order to confirm that caspases activation was involved in 10-acetylirciformonin B-induced cancer cell death, HL 60 cells were pretreated with 20 nM of caspase 8 or 9 inhibitors for 2 h and then treated with 1.25 or 2.5 g/mL of 10-acetylirciformonin B for 24 h. Pretreatment with caspase 8 or 9 inhibitors attenuated the effect of 10-acetylirciformonin B by 13% and 27%, respectively (Figure 4C). The data suggest that 10-acetylirciformonin B-induced apoptosis may involve activation of caspase 8 or 9.

## 3. Experimental

### 3.1. Bioassays Materials

RPMI 1640 medium, foetal calf serum (FCS), trypan blue, penicillin G, and streptomycin were obtained from Gibco BRL (Gaithersburg , MD, USA). 3-(4,5-Dimethylthiazol-2-yl)-2,5-diphenyltetrazolium bromide (MTT) and dimethylsulfoxide (DMSO) were purchased from Sigma-Aldrich (St. Louis, MO, USA). Antibodies against caspases 3, 8 and 9, -H2A.X and p-CHK2 (Ser68), were purchased from Cell Signalling Technologies (Beverly, MA, USA). Antibodies of Bcl-x, Bax, PARP, GAPDH and all other chemicals were obtained from Santa Cruz Biotechnology (Santa Cruz, CA, USA). Anti-mouse and rabbit IgG peroxidase-conjugated secondary antibody were purchased from Pierce (Rockford, IL , USA). Hybond ECL transfer membrane and ECL Western blotting detection kits were obtained from Amersham Life Sciences (Amersham, Buckinghamshire, UK).

### 3.2. Preparation of 10-Acetylirciformonin B

10-Acetylirciformonin B (Figure 1) was isolated and purified from marine sponge, and its chemical structure was identified by interpretation of spectral data (^1^H-NMR, ^13^C-NMR, and 2D NMR) as previously described [20]. This compound was dissolved in DMSO at a concentration of 20 g/mL and diluted before used.

### 3.3. MTT Antiproliferative Assay

Cells were seeded at 4 × 10^4^ per well in 96-well culture plates before treatment with different concentrations of the tested compound. After treatment for 24, 48, or 72 h, the cytotoxicity of the tested compound is determined using an MTT cell antiproliferative assay. Light absorbance values (OD = OD_570_ − OD_620_) were recorded at wavelengths of 570 and 620 nm using an ELISA reader for calculating the 50% inhibitory concentration (IC_50_), *i.e.*, the cell concentration at which the light absorbance value of the experimental group is one half of the control group. These results were expressed as a percentage of the control ± SD established from n = 4 wells per one experiment from three separate experiments.

### 3.4. Annexin V/PI Apoptosis Assay

The externalization of phosphatidylserine (PS) and membrane integrity were quantified using an annexin V-FITC staining kit. In brief, 10^6^ cells were grown in 35 mm diameter plates and were labeled with annexin V-FITC (10 µg/mL) and PI (20 µg/mL) prior to harvesting. After labeling, all plates were washed with binding buffer and harvested. Cells were resuspended in the binding buffer at a concentration of 2 × 10^5^ cells/mL before analysis by flow cytometry.

### 3.5. Neutral Comet Assay for Detection of DNA Double-strand Breaks (DSBs)

The assay was carried out using a CometAssay^TM^ Kit (Trevigen, Gaithersburg, MD, USA) following the manufacturer’s protocol for the neutral Comet assay. Briefly, cancer cells (2 × 10^5^ cells/mL) were treated with 10-acetylirciformonin B at the indicated concentrations. Cells were combined with 1% low melting point agarose at a ratio of 1:10 (v/v) and immediately 75 μL of the mixture was pipetted onto CometSlide^TM^ and allowed to set at 4 °C in the dark. The slides were immersed in ice-cold lysis solution (Trevigen) for 30 to 60 min. The slides were placed in a horizontal electrophoresis apparatus and electrophoresed in 1X TBE (90 mM Tris-HCl, 90 mM boric acid, and 2 mM EDTA, pH 8.0) at 20 V for 10 min. The samples were then fixed in 70% ethanol and dried before stained with 1:10,000 SYBR Green I (Trevigen) to visualize cellular DNA. The fluorescence images were analyzed using the TriTek Comet Image program to circumscribe the “head” and the “tail” regions of each comet and the integrated fluorescence values of each defined area were recorded. The comet length was measured from the trailing edge of the nucleus to the leading edge of the tail. This length was indicative of the extent of DNA damage. Calculations were averaged per replicate.

### 3.6. Western Blotting Analysis

Cell lysates were prepared by treating the cells for 30 min in RIPA lysis buffer, 1% Nonidet P-40, 0.5% sodium deoxycholate, 0.1% sodium dodecyl sulphate (SDS), 1 mM sodium orthovanadate, 100 µg/mL phenylmethylsulfonyl fluoride and 30 µg/mL aprotinin) (all chemicals were from Sigma) [21]. The lysates were centrifuged at 20,000 × *g* for 30 min, and the protein concentration in the supernatant was determined using a BCA protein assay kit (Pierce). Equal amounts of proteins were respectively separated by 7.5%, 10% or 12% of SDS-polyacrylamide gel electrophoresis and then were electrotransferred to a PVDF membrane. The membrane was blocked with a solution containing 5% non-fat dry milk TBST buffer (20 mM Tris-HCl, pH 7.4, 150 mM NaCl and 0.1% Tween 20) for 1 h and washed with TBST buffer. The protein expressions were monitored by immunoblotting using specific antibodies. These proteins were detected by an enhanced chemiluminescence kit (Pierce).

### 3.7. Statistics

The results were expressed as mean ± standard deviation (SD). Comparison in each experiment was performed using an unpaired Student’s *t*-test and a *p* value of less than 0.05 was considered to be statistically significant.

## 4. Conclusions

To the best of our knowledge, this is the first study to show that 10-acetylirciformonin B, a natural marine sponge furanosesterpenoid, inhibited the growth of HL 60 cells by activating the apoptotic mechanism and inducing DNA damage, as well as increasing p-CHK2 and -H2A.X expression. Treatment of 10-acetylirciformonin B triggered cell apoptosis through activation of caspases 8, 9, 3 which led to PARP cleavage as well as the down-regulation of Bcl-x and the up-regulation of Bax. On the other hand, pretreatment with caspase 8 or 9 inhibitors decreased the antiproliferative effect of 10-acetylirciformonin B, revealing that caspases activation was involved in the apoptotic effects of this compound. The cytotoxic results of 10-acetylirciformonin B provide a new insight into the potential use of this compound as a novel DNA damaging agent with minimum side effects. 
